# Comparative evaluation of bone marrow and dental pulp mesenchymal stem cells for motor functional recovery in rat sciatic nerve injury

**DOI:** 10.1111/jcmm.18340

**Published:** 2024-04-29

**Authors:** Elgin Orçum Uzunlu, Zeki Oğurtan

**Affiliations:** ^1^ Surgery Department, Faculty of Veterinary Medicine Selcuk University Konya Turkey

**Keywords:** laboratory animals, neurosurgery, stem cell therapy

## Abstract

This study delves into the impact of mesenchymal stem cells derived from bone marrow (BM‐MSCs) and those sourced from dental pulp (DP‐MSCs) on the recovery of motor function and morphological aspects of the rat's sciatic nerve after crush injuries. The findings highlight that the groups treated with BM‐MSCs, DP‐MSCs or a combination of both (BM + DP‐MSCs) displayed enhanced sciatic functional index values when juxtaposed with the sham group. This points to bettered motor functionalities. A deeper morphological analysis showed that all the groups had retained perineurium structure and fascicular arrangement. Notably, the sham and BM‐MSCs groups had very few inconsistencies. All groups showed standard vascular density. Remarkably, the combined treatment group (BM + DP‐MSCs) presented diminished oedema and a lower count of inflammatory cells. Through immunohistochemical methods, the presence of S100 expression was noted in the groups that underwent treatment. In summation, the study suggests that both BM‐MSCs and DP‐MSCs, whether used singly or in combination, can significantly aid in motor function restoration and morphological enhancements. An interesting observation from our research and earlier studies is that stem cells from dental pulp, which are sourced with less discomfort from milk and wisdom teeth, show a heightened propensity to evolve into nerve cells. This is in contrast to the more uncomfortably acquired BM‐MSCs.

## 1 INTRODUCTION

Stem cells are non‐specialized cells that can renew themselves, have the ability to divide unlimitedly and can transform into more than one cell outside of their own origin when the body needs it.^1^ Mesenchymal stem cells (MSCs) are defined as multipotent stem cells that can transform into mesenchymal tissue cells such as bone, cartilage, tendon, and muscle in vitro and in vivo.^2^ The easy isolation of MSCs, their reproduction in culture and high potential to spread ex vivo make these cells clinically valuable in both cellular and gene‐level treatments.^3^

Mesenchymal progenitor cells show the ability to produce interleukins and chemokines by synthesizing a number of growth elements, cytokines, chemokines and extracellular matrix proteins with and without haematopoietic origin.^4^ It has been fuelled by the findings that it does not express (or at very low levels) class I antigens and has only low expression of MHC class II antigens.^5^ In addition, they lack the co‐stimulatory molecules of the B7 family, which are necessary to initiate an immune response. This unique feature allows MSC preparations to cross MHC barriers without the concern of immunological rejection and the need for immunosuppression, which makes MSC a universal source of stem cells.^5^

In this study, we investigated the effects of rat bone marrow‐derived mesenchymal stem cells (BM‐MSCs) and rat dental pulp‐derived mesenchymal stem cells (DP‐MSCs) on the hind leg motor functional recovery and morphological parameters of the sciatic nerve in rats damaged by crushing injury.

## 2 MATERIALS AND METHODS

The study specimen consisted of 32 male Wistar Albino rats, 12–24 weeks old, weighing 250–300 g, obtained from the Experimental Medicine Application and Research Center of Selcuk University. Four rats were used to obtain MSCs from dental pulps and bone marrows, and 28 of them was used to form four randomly selected experimental groups, each having seven, for the sciatic nerve damage. The rats' left feet were used as a positive control since no procedure was performed on them.

In the experimental groups, the sciatic nerve in the right hind legs of all rats was exposed and subjected to crush injury. Subsequently, in the respective groups, Isotonic Solution, BM‐MSCs, DP‐MSCs and a combination of BM‐MSCs + DP‐MSCs were administered only once. After the injury, the first group (sham) received a single dose of 0.25 mL of 0.9% NaCl saline, the second group received 1 × 10^6^ BM‐MSCs, the third group received 1 × 10^6^ DP‐MSCs and the fourth group was given a combination of (0.5 × 10^6^ BM‐MSCs) + 0.5 × 10^6^ DP‐MSCs directly to the injured area.

The same anaesthesia protocol was used for all the rats. In order to obtain BM‐MMSCs and DP‐MSCs, rats were placed under general anaesthesia by administering appropriate doses of xylazine HCl (5–13 mg/kg, IM, Intermed Ecza Deposu, Turkey) and ketamine HCl (40–87 mg/kg, IM, Richter Pharma AG, Austria) and then euthanized by cervical dislocation. BM‐MSCs were isolated from bone marrow in a similar manner as previously described^6^ and DP‐MSCs were isolated from dental pulp of incisors similar to that previously described.^7^

Nervus ishiaducus were exposed under general anaesthesia, through sterno‐abdominal position on the lateral regions of the right femur. A pressure‐compression force of 1.5 g/mm^2^ was applied to the 2 mm part of the nervus ishiadicus, 1 mm away from the internal obturator canal, 10 mm proximal to the trifurcation, with the help of a B‐3V vessel clamp (S&T Marketing Ltd, Neuhausen, Switzerland) for 20 min and accordingly with resultant of ischaemia. Marking was applied from the epineurium with polypropylene 9‐0 surgical thread, 1 mm proximal to the damaged area. Afterwards, BM‐MSCs from the bone marrow and DP‐MSCs from the dental pulp origin and saline were applied to the injured site in the related rats. The skin and subcutaneous tissues of all rats were closed by paying attention to homeostasis. Flunixin meglumine (2.5 mg/kg, SC, Alke, Turkey) was administered as a post‐operative analgesic. For prophylaxis, cefazolin (15–25 mg/kg, SC, Ibrahim Ethem, Turkey) was administered every 24 h for 5 days. Animals were kept alive for 4 weeks at 21 ± 1°C room temperature, with a 12‐h dark–light cycle and fed ad libitum. Twenty‐eight days later, the animals were given high‐dose general anaesthesia of xylazine HCl + ketamine HCl, below 10 and 90 mg/kg, IM, respectively, and euthanasia was performed with cervical dislocation and the treated 10‐mm section of the nervus ishiaducus was removed. A 10‐mm section of intact and nontreated ishiaducus was removed for histopathological and S100 immunohistochemical staining from the left of the six rats as described by Carriel.^8^

### 2.1 Evaluation methods

**FIGURE 1:**
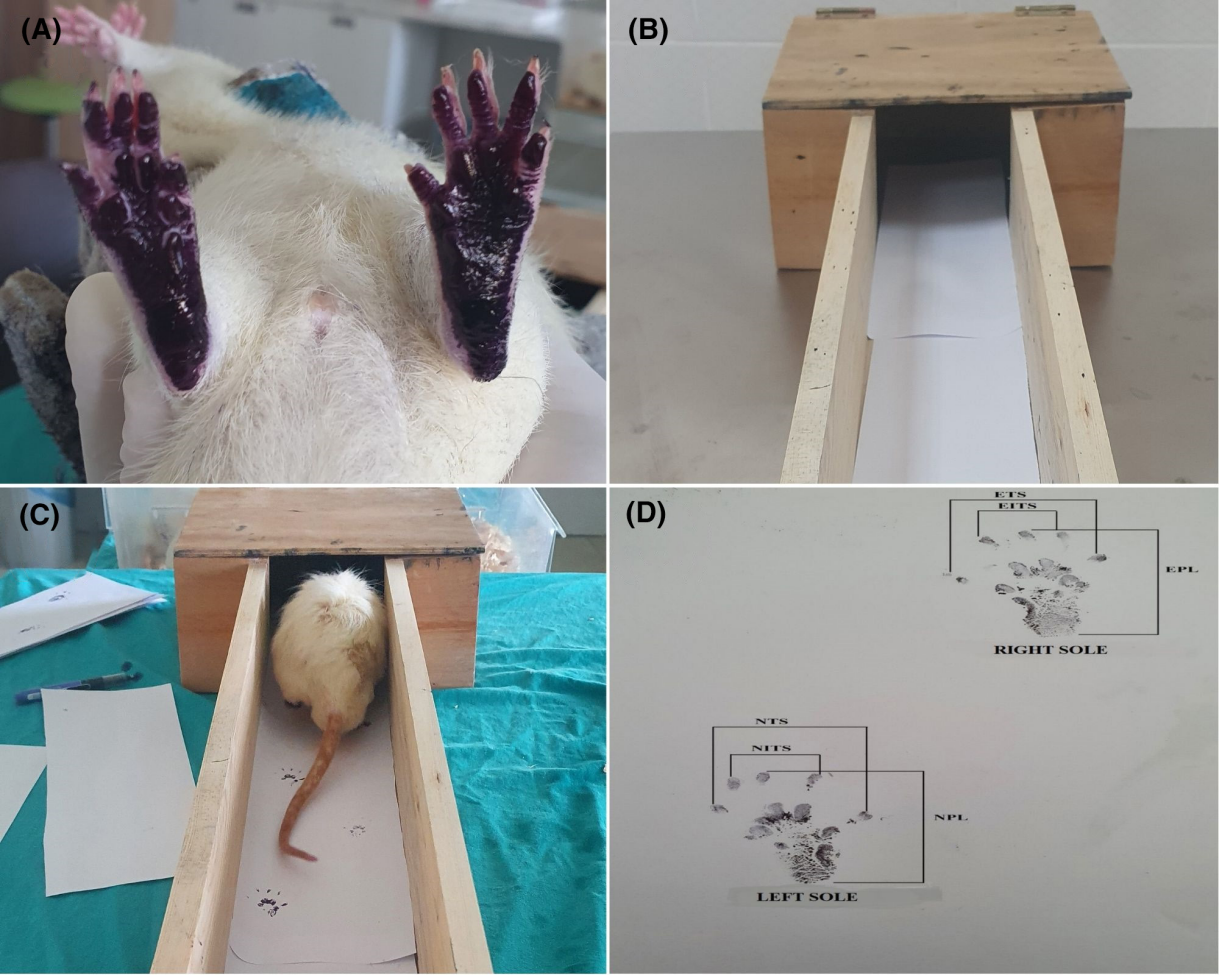
Execution of the rats for sciatic functional index (SFI). (A) Painting the underfoot of the rats; (B) walking corridor; (C) footprints formed following walking; (D) footprints of the soles of the feet for SFI measurement.

In terms of evaluation of application effects for the motor recovery, animals were first subjected to foot‐print testing on days 7, 14, 21 and 28 (Figure 1), and the motor functions of the animals were evaluated according to their walking movements on the paper.

Anti‐S100 immunohistochemical (pan 2008) and H&E (pan 2008) staining were carried out as previously described. Anti‐S100 immunohistochemical and H&E stained preparations were examined under the light microscope (Olympus BX51, Tokyo, Japan) and photographed (Olympus E‐PL3, Japan).

The digital images were analysed using computer‐based morphometry. The area of the entire nerve specimen in each image was selected and outlined for analysis using a pen tool for all the sections. The artefacts were excluded from the analyses using a negative pen of the ImageScope viewing software manipulated by two independent pathological experts who were unaware of the section origins. The measurement of each image was repeated three times for statistical analysis. According to the automatically calculated parameters, the percentage of the positive and strong pixels to total stained pixels (%) was determined. The stained area (%) for S100 immunohistochemistry was measured.

### 2.2 Calculation of sciatic nerve function index

The sciatic functional index (SFI) of the rats was measured on the 7th, 14th, 21st and 28th days after the surgery to control the healing of the damaged nerves. The soles of the hind feet of the rats were painted with black ink, and then, the rats were led on a white paper through a wooden corridor leading to a dark box with a width of 8 cm, length of 40 cm and height of 15 cm, in order to walk in a straight direction and the traces of the normal and treated feet were photographed (OLYMPUS E‐PL3, Japan) and SFI values were calculated for each rat (Figure 1D).

### 2.3 Statistical analysis

Comparisons between groups were made with one‐way variant analysis (ANOVA). Pairwise comparisons were made with the Post Hoc Test, Tukey HSD. The significance level was accepted as 0.05.

## 3 RESULTS

### 3.1 Surface markers results

**FIGURE 2:**
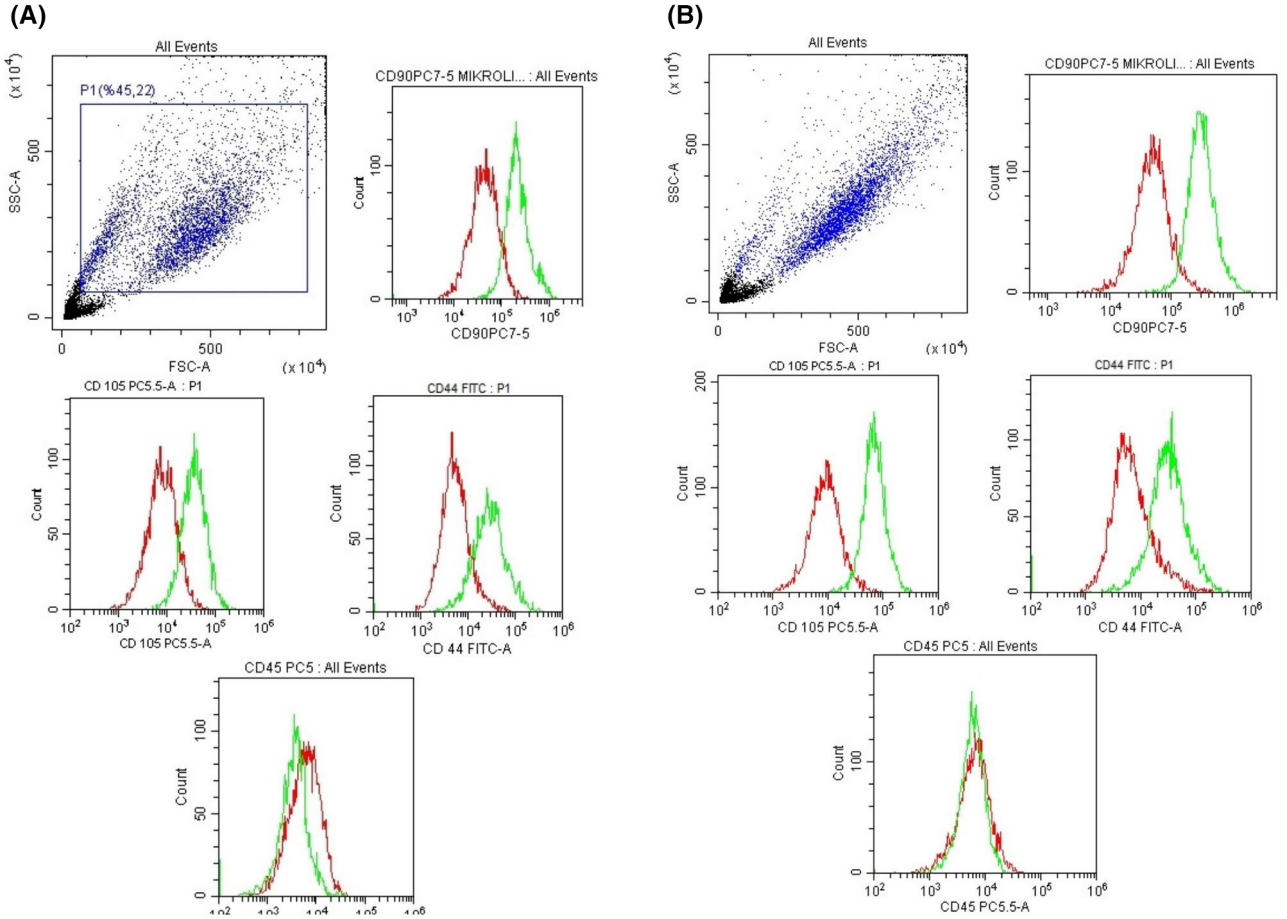
Flow‐cytometry results of bone marrow‐derived mesenchymal stem cells and dental pulp‐derived mesenchymal stem cells. (A) CD90^+^, CD105^+^, CD44^+^ and CD45^−^ surface markers; (B) CD90^+^, CD105^+^, CD44^+^ and CD45^−^ surface markers.

In our study, bone marrow‐derived stem cells dental pulp‐deriver stem cells was characterized by CD90^+^, CD105^+^, CD44^+^ and CD45^−^ by flow‐cytometric analysis (Figure 2).

### 3.2 General physical findings

During the study, care was taken with ad‐libitum feeding and sheltering methods, and daily incisional wound follow‐up and dressing were performed in the first week. No death was observed in the experimental groups but loss of fingers due to paralysis was observed after 8 days in two animals (Group 1: in rat 1, second and third fingers; in rat 6, second, third and fourth fingers). These animals at 14, 21 and 28 days were not included in the gait analysis. It was observed that the rats in the first sham group walked on their heels until the third week and then on the soles of their feet, while in other groups they walked on the soles of their feet after the second week.

### 3.3 General histomorphological

**FIGURE 3:**
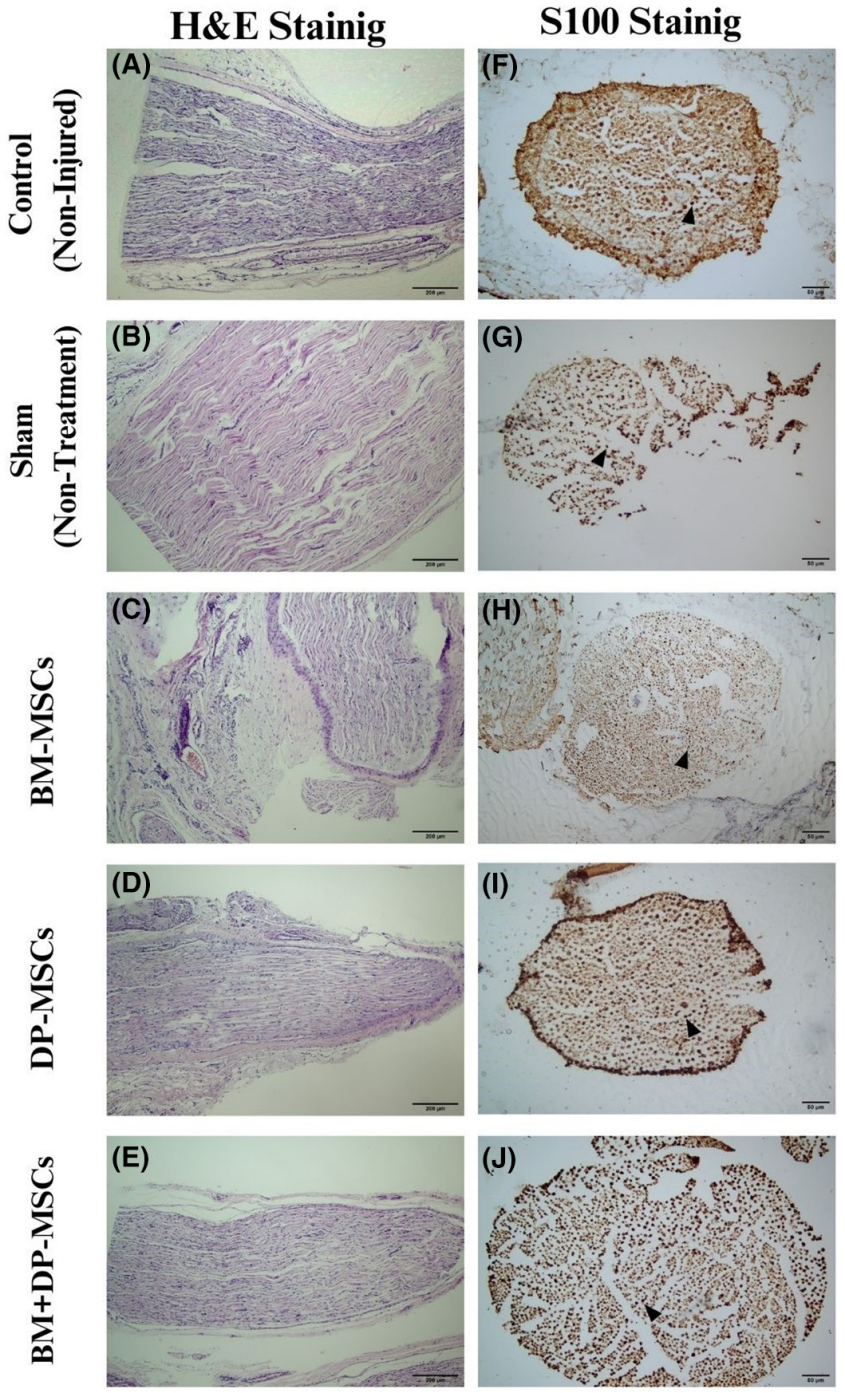
H&E (bar = 200 μm) and S100 (bar = 50 μm) results: (A) control tissue stained with H&E; (B) sham tissue stained with H&E; (C) BM‐MSC tissue stained with H&E; (D) DP‐MSC tissue stained with H&E; (E) BM + DP‐MSC tissue stained with H&E; (F) control tissue stained with anti S100; (G) sham tissue stained with anti S100; (H) BM‐MSC tissue stained with anti S100; (I) DP‐MSC tissue stained with anti S100; (J) BM + DP‐MSC tissue stained with anti S100.

Perineurium, fascicle organization of the nerve, vascular density, oedema, presence and density of inflammatory cells, and necrosis/degeneration were evaluated with H&E staining (Figure 3A–E).

The perineurium structure was preserved in all groups of rats. The fascicular organization was observed to be preserved in all groups, except in groups 1 and 2, which was minimally irregular. Vascular density was found to be normal in all groups. While the presence of oedema was observed in groups 1, 2 and 3, there was none in groups 4 and 5. There was a slight increase in the density of inflammatory cells in all the groups of rats except control group 5, which was absent in all the rats. While there was mild focal necrotic degeneration in groups 1 and 2, there was none in groups 3, 4 and 5.

### 3.4 Immunohistochemical findings

The percentage and intensity of S100 staining (Figure 3F–J, Table 1) was accepted as 100% for the control cases, and the rate in other cases was evaluated accordingly. S100 expression was not observed in any of the animals in group 1 – sham single administration group. Rates of 70% in the second‐BM‐MSCs, and 90% in the third‐DP‐MSCs and the fourth‐BM + DP‐MSCs and 100% in the fifth‐control groups were observed in all rats.

**TABLE 1:** S‐100 immunochemistry results.

Group	Animal	S‐100 staining percentage
Control	Rat 4	100
	Rat 5	100
	Rat 6	100
Sham	Rat 5	–
	Rat 6	–
	Rat 7	–
BM‐MSCs	Rat 4	70
	Rat 5	70
	Rat 6	70
DP‐MSCs	Rat 4	90
	Rat 5	90
	Rat 6	90
BM + DP‐MSCs	Rat 4	90
	Rat 5	90
	Rat 6	90

### 3.5 Gait analysis and sciatic function index findings

**FIGURE 4:**
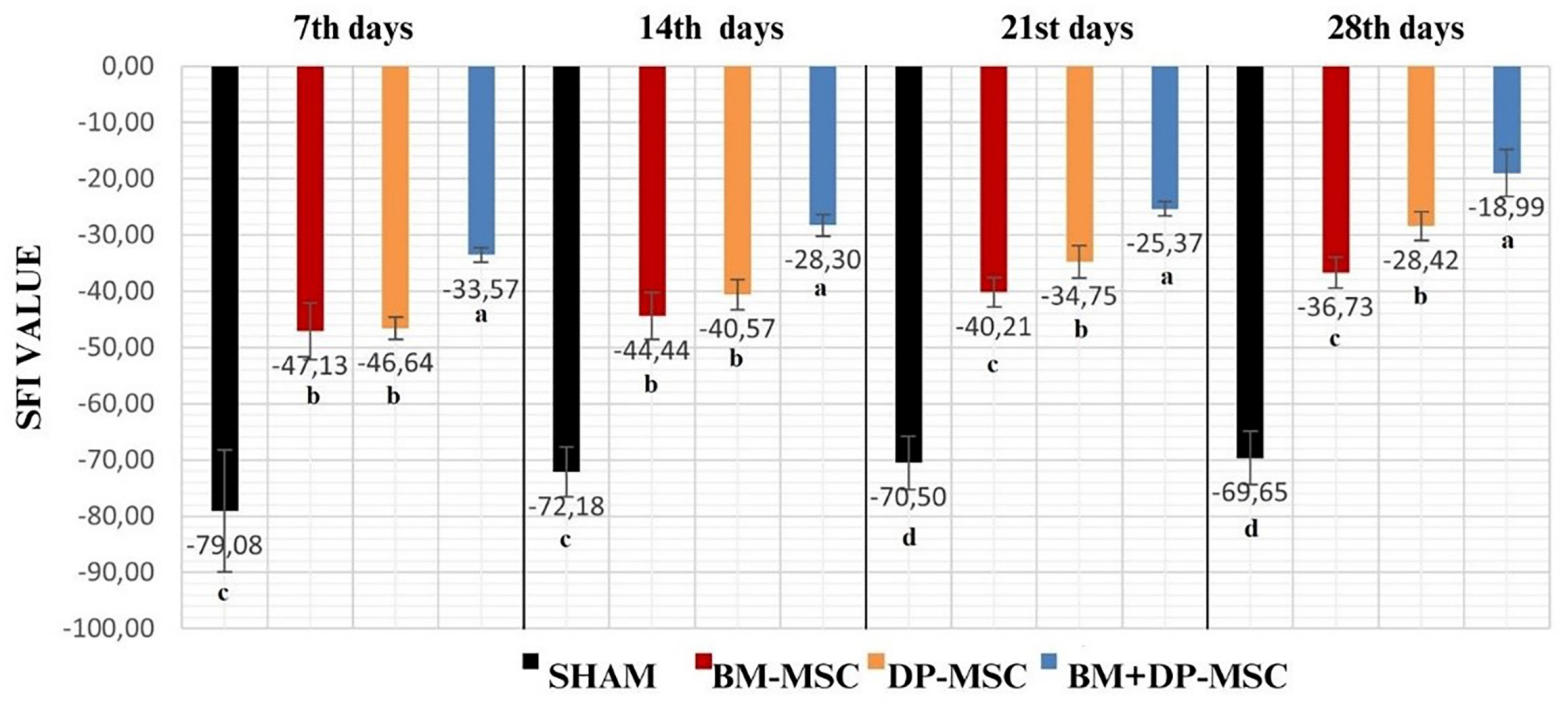
Weekly sciatic functional index results of the first, second, third and fourth groups (*p* < 0.05).

Gait analysis of rats in all the groups was performed on days 7, 14, 21 and 28. The SFI results were shown in Figure 4. Walking test analysis could not be performed in two rats (rats 1 and 6) in the first‐sham group, and in the second‐BM‐MSCs group in one rat (rat 3) due to the absence of toes after the first week.

### 3.6 Comparison of the first and second week SFI results

The same identical results were found in the first and second weeks for all groups as follows: Group 4 BM + DP‐MSCs SFI values were found to be significantly increased compared to other groups (*p* < 0.05). While the results of group 2 BM‐MSCs and group 3 DP‐MSCs SFI were not statistically different from each other, group 2 BM‐MSC and group 3 DP‐MSC SFI values were found to be statistically increased compared to the first sham group values (*p* < 0.05) (Figure 4).

### 3.7 Comparison of the third and fourth week SFI results

The same identical results were also found in the third and fourth weeks for all groups as follows: Group 4 BM + DP‐MSCs, group 3 DP‐MSCs and group 2 BM‐MSCs SFI values were found to be significantly (*p* < 0.05) increased compared to that of group 1 in the third and fourth weeks, which means that the improvement was better in these three groups compared to the first group (Figure 4). There was a statistically significant difference (*p* < 0.05) in the following order among the groups, in favour of groups 4, 3 and 2 in the third and fourth weeks.

### 3.8 Comparison of the group 1 sham SFI results

Analysis of the results of the 7th, 14th, 21st and 28th days revealed no statistically significant difference for the sham groups among the related days (Figure 4).

### 3.9 Comparison of the group 2 BM‐MSC SFI results

There was no statistically significant difference between the SFI results of the 28th and 21st days, and between the 14th and 7th days, and 21st and 14th days in the group 2 BM‐MSCs, but the 28th‐day results increased significantly from that of the 7th and 14th days (*p* < 0.05). It was found that the SFI value of the 21st and 28th day results increased significantly (*p* < 0.05) compared to that of the first week. It was observed that the improvement in BM‐MSCs was statistically more effective after the 21st day (Figure 4).

### 3.10 Comparison of group 3 DP‐MSC and group 4 BM + D‐PMSC SFI results

When group 3 DP‐MSC and group 4 BM + DP‐MSC SFI results were evaluated, it was found that the results of the 28th day were significantly (*p* < 0.05) increased compared to those of the 7th, 14th and 21st days. The difference among the day groups was statistically significant (*p* < 0.05) in the following order, in favour of 28th, 14th and 7th days (Figure 4).

## 4 DISCUSSION

In our study, second BM‐MSCs, third DP‐MSCs and fourth BM + DP‐MSC application groups showed a continued increased SFI values starting from the 7th day and thus the ongoing improvement as far as clinical healing is concerned based on the gait analyses, which is consistent to the findings of Marconi et al.^9^ that, although they used 2 × 10^6^ twice the amount of human‐derived adipose tissue MSC (AD‐MSC) stem cells from the tail vein for 30‐s‐applied‐Dumont Jewellers clamp injured ishiaducus nerve in mice that we used in our study. Among the groups in our study, there was also no difference with no improvement between the 1st and 4th week for the sham group SFI values, which is parallel with that of Marconi et al.^9^

In their study on rats, Yang et al.^10^ applied a pressure‐compression force of 1.5 g/mm^2^ for 20 min and created ischaemia in the sciatic nerve of rats by using of a B‐3V vascular clamp. In their study, they compared 1 × 10^5^ rat bone marrow‐derived MSC alone and in combination with cold water swimming (MSCCWS) at an average of 16.5°C after crush injury. They used one‐tenth of the stem cells that we used in our study. They found significantly lower SFI values, indicating significantly more favourable recovery rate in the MSC‐CWS group compared to other groups at 4 weeks which is in accordance with the results obtained in our study. Our SFI results at the first week in groups BM‐MSC (−47.13), DP‐MSC (−46.64), BM + DP‐MSC (−33.57) and at the fourth week in groups BM‐MSC (−36.73), DP‐MSC (−28.42), BM + DP‐MSC (−18.98) is obvious to be far better as far as functional motor activity is concerned compared to their even combined use of cold swimming effect for SFI results at the first and fifth postoperative week as being ≌−80 and ≌−40, respectively in the MSCCWS in the study of Yang et al.^10^

The high rate of S100 expression as an important indicator of recovery in all groups except the sham group in our study which is in parallel with the findings of Yang et al.^10^ Although similar results were reported by Yang et al.^10^ they used one‐tenth of the stem cells that we used in our study. Obtaining the same results with one‐tenth of the stem cells might be due to the affirmative effect of swimming factor.

It should be taken into account that using differentiated stem cells may reduce its own potential in its use by differentiating into a known cell with limited effect and degree of influence whether useful or not. On the other hand, it may be more effective and advantageous to use the undifferentiated stem cells without differentiation, since it is the most effective in its niche and transforming into a required different cell or cells rather than to a known cell with a limited effect. Moreover, comparing differentiated and undifferentiated stem cells will be important in terms of clarifying this issue.

In their study on rats, Yang et al.^11^ applied a pressure‐compression force of 1.5 g/mm^2^ with the help of a B‐3V vascular clamp in the rat nervus ishiadicus, for 20 min, after which treated with using 1 × 10^5^ bone marrow‐derived rat MSCs given to the epineurium layer and in combination with other modalities. Yang et al.^11^ used one‐tenth of the stem cells that we used in our study. They compared MSC, low‐level laser therapy (LLLT) and the combination of these two (MSCLLLT). In their study, they measured SFI values 12 hours before the operation and at 7, 14, 21 and 28 days after the operation. Their seventh‐day SFI results as of MSCLLLT (−74.6 ± 5.0), MSC (−86.8 ± 8), LLLT (−80.0 ± 4.1) and sham (−91.3 ± 12.2), 14th‐day SFI results as of MSCLLLT (−37.0 ± 3.5), MSC (−74.5 ± 8.7), LLLT (−68.7 ± 5.2) and sham (−90.0 ± 3.9), 21st‐day SFI results as of MSCLLLT (−29.0 ± 3.1), MSC (−58.4 ± 5.4), LLLT (−55.3 ± 4.6) and sham (−75.3 ± 3.0), 28th‐day SFI results as of MSCLLLT (−24.0 ± 3.1), MSC (−50.4 ± 4.8), LLLT (−51.6 ± 3.8) and sham (−69.9 ± 2.8). In our study of all groups at the 7th day, SFI results (Figure 4) was far better than those of SFI above of Yang et al.^11^ Only combined use of MSCLLLT SFI results above of Yang et al.^11^ was lower in their study compared to those of SFI in the BM‐MSC and DP‐MSC groups at the 14th, 21st and 28th days. On the other hand in the BM + DP‐MSC SFI results at the 7th, 14th, 21st and 28th days in our study (Figure 4) was lower with more favourable walking performance compared to that of SFI results of even the combined use of MSCLLLT above of Yang et al.^11^

This low rate is thought to be due to the fact that the number of stem cells we used in our study was 10 times higher. It is obvious that increased number of cells (10 times in our study) seems to result in eliminating the use of second tool as a combined therapy as cold swimming,^15^ and low‐level laser therapy^11^ would be easier and practical as far as comfort of the patient and time and expenses are concerned. In addition to this obtaining better results in our study in contrast to those of Yang et al.^11^ Yang et al.^11^ also indicates the importance of using higher number of cells.

In our study, we observed that the presence and intensity of inflammatory cells were higher in the sham (slightly), BM‐MSC (slightly), DP‐MSC (slightly) and BM + DP‐MSC (slightly) groups compared to the non‐damaged tissue in the H&E stained of the damaged nerve tissue harvested after 4 weeks of nerve crush injury. Consistent with our findings, Yang et al.^11^ also observed an increase in the presence and intensity of inflammatory cells in the MSCLLLT, LLLT and MSC groups compared to non‐damaged tissue.

After nerve injury, an increase in the presence and intensity of inflammatory cells, particularly macrophages, indicates the occurrence of the inflammatory process. In the damaged tissue, this process occurs as inflammatory cells migrate to the damaged area in an attempt to clean up the damaged tissue fragments.^12^ This process is important for the renewal and healing of cells in the damaged area. Therefore, controlling the presence and intensity of inflammatory cells is one of the significant parameters indicating tissue healing.

Using a slightly different method than that of in our study, Yang et al.^11^ marking and calculating S100‐stained area in two different computer applications found that the percentages of S100 staining were 40% in non‐treated, 50% both in MSC and LLLT, and more than 60% in MSCLLLT groups. They performed statistical analysis using Scheffé's post hoc test and did not find a significant difference between the MSC and LLLT groups, but found that the MSCLLLT group was significantly higher than the non‐treated MSC and LLLT groups. In our study, we obtained cell density 70% in the BM‐MSC, 90% in the DP‐MSC and 90% in the BM + DP‐MSC groups, with no results in the sham group. It was not possible to make a direct comparison due to the different evaluation methods and the approximate percentage values provided by Yang et al.^11^ however, it is a common finding that S100 staining increased in both studies after treatment.

Ma et al.^13^ had 30 s crush injury with the help of No. 5 Dumont Jewellers clamp, and treated it with differentiated MSC extracellular vesicles (MSCEV) from the rat bone marrow and reported significant improvement of SFI values in the MSCEV from the first week (≌−80), second week (≌−70), third week (EVs ≌−40) to the fourth week (EVs ≌−30). All groups in our study had lower SFI values (Figure 4) compared to the first, second and fourth week results of MSCEV. Only BM‐MSC group in our study at the third week had almost identical SFI value of ≌−40 as in the Ma et al.^13^

Okuwa et al.^14^ applied 0.59 N pressure‐compression force for 30 min with the help of a clamp in the ishiaducus nerve in rats, used dental pulp MSC obtained from human molar teeth instead of rats in the treatment, and observed significant improvement from the 7th day to the 10th and 14th days based on the gait analyses. The SFI values of the BM‐MSC, the DP‐MSC and the BM + DP‐MSC groups in our study showed progressive improvement starting from the first week, which are consistent with the findings of Okuwa et al.^14^ at 10th and 14th day results.

Wang et al.^15^ placed a vessel clip on the sciatic nerve of rats and subjected it to a crushing force of 30 g for 2 min and used human dental pulp stem cells (DPSCs) in one group without exposing them to the differentiation process, and in another group used human dental pulp mesenchymal stem cells by applying neurogenic differentiation (N‐DPSCs). They did not provide information about the number of stem cells they used in their studies. No difference was observed among all groups 3 days after the crush injury. They did not provide any information about whether there was a significant difference in the statistical results of SFI values in the first week. They showed that2 weeks after the crush injury, both the DPSC group (−68.53) and the NDPSC (−38.73) group showed signs of recovery while the SFI of the control group (≌−80) had worsened. When we look at the results of the second week of our study, it is obvious that we obtained higher SFI values for the BM + DP‐MSC (−28.30) in our study compared to DPSC group (−68.53) and the NDPSC (−38.73) group in Wang et al.^15^ and for the BM‐MSC (−44.44) and DP‐MSC (−40.57) in our study groups compared to DPSC group (−68.53) in Wang et al.^15^ Three weeks after the operation, the SFI indicated that the motor function of the rats in the NDPSC group (≌−40) had a continuous recovery compared with that of the rats in the DPSC group (≌−60) and the control group (between −60 and −80), which tended to be stable 2 weeks after surgery but showed no inclination toward recovery (control group: −68.53 vs. NDPSC group: −38.73). In their study the differences between these three groups were obvious 4 weeks after operation (control group: −67.23 vs. NDPSC group: −40.08). When we look at the results of the fourth week of our study, it is seen that we obtained higher SFI values in our BM‐MSC (−36.73), DP‐MSC (−28.42) and BM + DP‐MSC (−18.99) groups compared to those groups in Wang et al.^15^

In parallel with our study, Wang et al.^15^ showed that implanted hDPSCs and NhDPSCs can help locomotor recovery after nerve crush injury, based on the SFI values of the three groups in their study. In their study contrary to our study the repair effects of the implanted DPSCs were only obvious in the first week and showed almost no effects in the later period. In our study, DPSCs showed improvement from the first week. In their study the implanted NhDPSCs presented sustained and stable effects of motor function recovery until the fourth week. They showed that the secretion of neurotrophic factors may contribute to recovery. In our study, BM‐MSCs, DP‐MSCs and BM + DP‐MSCs, which were used without differentiation, gradually improved from the first week to the fourth week. The reason for this effect is thought to be due to the fact that the use of undifferentiated stem cells naturally differentiates into the cells needed in the region depending on the niche.

Wang et al.^15^ in their study after 4 weeks used H&E staining to assess the level of deterioration and regeneration in the nerves. As a result, they found that the nerve fibres in the control group were irregular, discontinuous and sparse, whereas the number of nerve fibres in the DPSC and NDPSC groups was higher than the control group, and the nerve fibres of these two groups had clearer lines and were well organized. In their study, they looked at the level of regeneration of the myelin sheath by immunofluorescence staining method. Contrary to our study, they did not use s100 immunohistochemistry. In their study, NDPSCs had more pronounced effects on nerve remyelination, repair and regeneration.

Bucan et al.^16^ applied unknown cell number implantation of exosome and adipose mesenchymal cells (adMSC) following a crush injury of unknown pressure with No. 5 Dumont forceps to the sciatic nerve of adult Wistar albino rats for 10 s. Immediately following the injury, the SFI values were found to be (−88.58 ± 18.4), (−51.89 ± 18) (−67.72 ± 46.4) in the sham, adMSC and adMSCExosomes groups respectively. On day 7, they were (−51.81 ± 8.01), (−38.98 ± 5.36) and approximately (−50) in the Sham, ADMSC and ADMSCExosomes groups respectively. On day 14, they were approximately −40, (−32.20 ± 23.88) and approximately (−30) in the sham, adMSC and adMSCExosomes groups respectively. On day 21, they were (−19.07 ± 6.24), close to (0) and (−16.75 ± 1.64) in the sham, adMSC and adMSCExosomes groups respectively. Their SFI values revealed no statistically significant difference among the groups on a weekly basis. It was also observed that SFI values were higher in their study compared to those in our study; however, this discrepancy is believed to be due to the 19 min and 50 s difference in the duration of injury, keeping in mind that our crushing injury time was 20 min, far longer than 10 s of their application. Although their study demonstrated that exosomes triggered biological effects in target tissues through the transfer of genetic material and growth factor proteins, models treated only with adMSC compared to adMSCExosomes showed better but statistically non‐significant improvement.

Tremp et al.^17^ induced a crush injury to the left sciatic nerve using a polymer ligature clip for as long as 2 weeks in Sprague–Dawley rats. Two weeks later, an ultrasound‐guided injection was performed, applying 25 μL of culture medium (CM) consisted of Dulbecco's modified Eagle medium with 10% foetal bovine serum and 1% penicillin/streptomycin, containing, 0.5 × 10^6^ rat adipose‐derived stem cells (rASC) to the epineurium immediately distal to the ligature clip, kept in place in the 1st group as rASC. In the second group as CM, an injection of 25 μL CM alone was carried out, using ultrasound guidance at the same location with the ligature clip, kept in place as is in the first group. After ligature clip removal (CR), the same protocols used with ligature CR in first and second groups were repeated in the third as rASC CR and fourth as CM CR groups respectively. They calculated the SFI values, and postmortem imaging was conducted using a 3‐Tesla MRI machine. Additionally, fractional anisotropy, gastrocnemius muscle weight ratio MWR and histomorphometric analyses were performed as measures of fibre integrity.

The best SFI values were observed in the rASCCR (−80.1 ± 8.5, −72.1 ± 6.8 and −59.3 ± 7.5), and in the rASC groups (−88.4 ± 11.2, −70 ± 16.3 and −67 ± 9.8) at 14, 28 and 42 days respectively. The lowest results were found in the CM (−83.2 ± 11.2, −93.7 ± 11.7 and −81.2 ± 11.9) and the CMCR groups (−84 ± 15, −91.4 ± 16.7 and −82 ± 6.6) at 14, 28 and 42 days respectively. The study reported a statistically significant difference at 42 days between the rASC with CR group and the CM with CR group, while no significant difference was found between the rASC group and the rASC with CR group. Additionally, no statistically significant difference was observed between the CM group and the CM with CR group.^17^ Their reported SFI values were notably low at 14 and 28 days compared to those in our study. However, it is difficult to make a comparison in their study because the extent of the pressure exerted by the clip is unknown.

There is no consistency among researchers^9^, ^10^, ^14^, ^15^, ^16^, ^17^ to make a damage in the nerve tissue as related to the use of different force of power ranging from 0.19 to 0.59 Newton based on published papers and using the calculation formulations below.^9^, ^10^, ^11^, ^12^, ^13^, ^14^, ^15^, ^16^, ^17^ Therefore, it is difficult to make a real comparisons due to this factor, and obtaining different results might be due to this neglected factor. It should be better to standardize the force to be used in the nerve damage.

Clamp area was calculated according to the information received from the manufacturer in the bv3 clamp we used. The force is calculated by using the clamp area and the gravitational acceleration constant. 30 [gf] = 0.29 [N]. The force is calculated by using the clamp area and the gravitational acceleration constant. 1.5 [g/(mm^2^)] is provided by the manufacturer. *F* = f*A*g (*F* = force, *f* = compressive pressure force, *A* = area, *g* = gravitational acceleration) formula was used to calculate the force. *A* = 13.125 $\left[mm^{2}\right]$, *f* = 1.5 $\left[\frac{g}{mm^{2}}\right]$, *g* = 9.81 $\left[\frac{m^{2}}{s}\right]$.

$$1.5\;\left[\frac{g}{mm^{2}}\right]*13.125\;\left[mm^{2}\right]*9.81\;\left[\frac{m^{2}}{s}\right]=193.13\;\left[g*\frac{m^{2}}{s}\right]=0.19\;\left[N\right]$$

The 30 [gf] is equal to *F* = ma. 30 g = 30 gram, *m* = mass = 30 gram = 0.03 kg, *a* = acceleration = *g* = 9.81 $\left[\frac{m^{2}}{s}\right]$, *F* = Force [N]; $0.03\;\left[\mathrm{kg}\right]*9.81\left[\frac{m^{2}}{s}\right]=0.29\;\left[N\right].$

## AUTHOR CONTRIBUTIONS

**Elgin Orçum Uzunlu:** Conceptualization (equal); data curation (equal); formal analysis (equal); funding acquisition (equal); investigation (equal); methodology (equal); project administration (equal); resources (equal); software (equal); supervision (equal); validation (equal); visualization (equal); writing – original draft (equal); writing – review and editing (equal). **Zeki Oğurtan:** Conceptualization (equal); data curation (equal); formal analysis (equal); funding acquisition (equal); investigation (equal); methodology (equal); project administration (equal); resources (equal); software (equal); supervision (equal); validation (equal); visualization (equal); writing – original draft (equal); writing – review and editing (equal).

## FUNDING INFORMATION

This research was supported by Selcuk University Scientific Research Projects Coordinator (No: 19102013). The publication of this article was supported by the Scientific and Technological Research Institution of Turkey (TUB1).

## CONFLICT OF INTEREST STATEMENT

The author(s) declared no conflicts of interest with respect to the research, authorship and/or publication of this article.

## Data Availability

Data supporting the findings of this study are available by e‐mail from the corresponding author [EOU (elginorcum.uzunlu@selcuk.edu.tr)] upon reasonable request.
